# The Long‐Term Effect of Childhood Otitis Media on Speech‐in‐Noise Testing at Ages 9 and 13

**DOI:** 10.1002/lio2.70077

**Published:** 2025-01-15

**Authors:** Stefanie N. H. Reijers, Jantien L. Vroegop, Bernd Kremer, Marc P. van der Schroeff

**Affiliations:** ^1^ Department of Otorhinolaryngology‐Head and Neck Surgery Erasmus University Medical Center Rotterdam The Netherlands; ^2^ The Generation R Study Group Erasmus University Medical Center Rotterdam The Netherlands

**Keywords:** children, digits‐in‐noise, otitis media, speech reception thresholds

## Abstract

**Objective:**

To investigate the relationship between a history of otitis media (OM) in early childhood and speech reception thresholds (SRT) in later childhood, using the Dutch digits‐in‐noise (DIN) test at ages 9 and 13 years.

**Methods:**

This study was conducted within the Generation R study, a prospective birth cohort in Rotterdam, the Netherlands. Children underwent pure‐tone audiometry and DIN testing at ages 9 (2011–2015) and 13 (2016–2020) years. Regression analyses and a linear mixed model were used to examine associations between OM history and SRT in noise, accounting for repeated measurements within individuals.

**Results:**

At baseline (age 9 years), 2063 children were included with a mean SRT of −5.6 (SD = 2.0). At follow‐up (age 13 years), 3382 children were included with a mean SRT of −7.4 (SD = 1.4). A positive association was found between recurrent acute OM (RAOM) and DIN test outcomes, with an estimated coefficient of 0.55 (95% CI = 0.26, 0.84; *t*‐value = 3.70). Socioeconomic status and multilingualism did not significantly predict DIN test performance.

**Conclusion:**

A history of RAOM may potentially influence SRT outcomes in later childhood, with higher DIN scores observed in the RAOM group. While the effect sizes between the groups are small to moderate, these findings highlight the importance of considering the potential long‐term effects of OM in both clinical practice and future research. Further studies are needed to better understand these relationships.

**Level of Evidence:**

2 (cohort study).

## Graphical abstract

Our longitudinal study provides valuable insights into how recurrent acute otitis media (RAOM) in children can affect auditory processing in noisy environments—a crucial aspect of effective communication. These findings highlight the importance of considering the long‐term effects of RAOM in both clinical practice and future research.

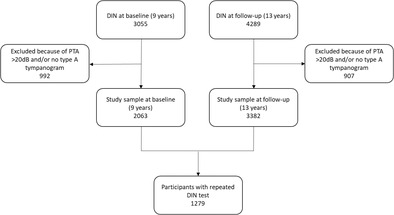

## 1 Introduction

Otitis media (OM) is a prevalent condition in childhood, manifesting in various forms, including acute otitis media (AOM), OM with effusion, recurrent AOM (RAOM), and chronic suppurative otitis media (CSOM) [1]. Understanding the potential long‐term ramifications of OM on hearing is critical, as it is the most common cause of hearing loss in children [2, 3]. Approximately 60% of children will experience at least one episode by age 3 years, with a quarter enduring three or more episodes [4]. The incidence of OM‐subtypes varies across populations due to factors such as social dynamics, demographics, and genetic predispositions [5].

The inflammatory process associated with OM can adversely affect hearing during the acute phase and may also compromise the integrity of the inner ear. Research indicates that the round window may be permeable to toxins and endotoxins [6, 7, 8]. Increased hearing thresholds within the high‐frequency range (specifically between 4 and 8 kHz) have been documented in individuals with RAOM [9, 10]. Additionally, studies have shown that altered afferent auditory input due to OM during childhood is linked to impaired function of descending neural pathways and poorer speech‐in‐noise recognition [11]. This underscores how disruptions in auditory input and language experience during early developmental stages can interfere with the maturation of auditory functions. However, the question of whether such disruptions lead to long‐term deficits remains debated in the literature.

Several studies have demonstrated persistent impairments in signal detection and discrimination in noisy conditions following the resolution of OM [12, 13, 14, 15, 16]. Notably, Okada et al. found that chronic conductive hearing loss was associated with speech intelligibility deficits in patients with normal bone‐conduction thresholds, suggesting that prolonged sound deprivation may adversely impact speech discrimination [8]. In this context, the work of Mishra et al. also provides important insights by investigating the impact of auditory deprivation during development on efferent neural feedback and perception. Although not exclusively focused on OM, their findings contribute valuable insights into the broader implications of early auditory challenges [17]. A critical review of the existing literature, including the comprehensive review by Whitton and Polley, highlights the need for further investigation into the mechanisms underlying the effects of OM on auditory function [18].

Recognizing this critical gap, we initiated a longitudinal study to explore the relationship between a history of OM and its potential effects on speech discrimination over time. The Generation R cohort, a large, longitudinal study designed to investigate a wide range of health outcomes, provides a unique opportunity to examine the impact of early life OM on auditory function, particularly speech discrimination. Through this research, we aim to offer nuanced insights into whether OM and its subtypes in early life affect long‐term speech perception.

## 2 Methods

### 2.1 Design

This study was embedded in the Generation R study, a population‐based prospective cohort study from early fetal life onwards in Rotterdam, the Netherlands [19]. The study recruited eligible expectant mothers with anticipated delivery dates falling between April 1, 2002 and January 1, 2006. Subsequent monitoring of both the mothers and their offspring involved the administration of regular surveys and physical examinations. Upon reaching 9 and 13 years of age, all participating children were extended invitations to the research facility at the Erasmus MC Sophia Children's Hospital in Rotterdam for an extensive physical evaluation, which included audiometric assessments (pure‐tone audiometry, speech‐in‐noise testing, and tympanometry), as well as the completion of standardized questionnaires regarding OM history. There were no data available on received ear surgery or hearing aids. A total of 2063 children were enrolled in this study at the age of 9 (baseline), and a total of 3382 at the age of 13 (follow‐up). Children meeting the criteria of having hearing thresholds of 20 dB hearing level (HL) or better at octave frequencies ranging from 250 to 8000 Hz and exhibiting normal type A tympanograms were included [20]. The 20 dB HL threshold was chosen to include only children with hearing thresholds within the range where normal auditory function would be expected, ensuring that participants did not have even mild hearing loss that could affect speech perception. Additionally, normal tympanograms were required to indicate healthy middle ear function, minimizing the risk of conductive hearing loss.

### 2.2 Pure‐Tone Audiometry

HLs were assessed in a soundproof cabin following ISO 8253‐1 standards using a Decos audiometer and TDH‐39P earphones. Calibration followed ISO 389‐1, with checks every 6 months. Pure‐tone air conduction thresholds were measured at 0.5, 1, 2, 3, 4, 6, and 8 kHz using the abbreviated rising method. Thresholds were defined by the intensity level at which the tone was heard in two out of three rises, with a 5 dB HL variance.

### 2.3 Speech‐in‐Noise Audiometry

Speech‐in‐noise testing was performed using the Dutch digits‐in‐noise (DIN) test with 70 dB stationary noise and digit triplets presented to the superior ear. The signal‐to‐noise ratio (SNR) was adjusted adaptively to determine the speech reception threshold (SRT) at a 50% correct response rate [21]. The initial triplet was presented at −10 dB SNR and adjusted in 2 dB steps based on correct or incorrect responses. The final SRT and standard deviation were automatically calculated from the last 20 triplets.

### 2.4 Tympanometry

Tympanometry was conducted with an Interacoustics AT235h tympanometer at 226 Hz. Measurements of ear canal volume, static compliance, and middle ear pressure were recorded. A standard tympanogram required ear canal volume > 0.3 mL, static compliance > 0.25 mL, and middle ear pressure between −100 and +100 daPa [22]. Any deviation from these criteria was classified as abnormal.

### 2.5 Otitis Media

Caregiver‐reported data on OM history were collected via questionnaires at 2, 6 months, annually from 1 to 4 years, and at 6 and 10 years. A diagnosis of AOM was based on the presence of one or more of the following criteria, each recorded as a yes/no response: fever and earache, earache combined with a doctor's visit, otorrhea (ear discharge), or the use of eardrops. For the younger children (e.g., at 2 and 6 months), the questionnaire focused on symptoms in the past few months. For older children (e.g., at 1, 3, 4 years), the caregiver‐reported history of OM covered the previous 12 months. The control group included children with two or fewer documented OM episodes and no OM in the past year. The OM group included children with two or more OM episodes by 3 years of age. The RAOM group included those with three or more episodes in 6 months or more than three episodes in 12 months, with at least one episode in the past 6 months. These criteria are commonly used to classify and differentiate AOM and RAOM cases in clinical studies [23].

### 2.6 Covariates

Participant characteristics encompassed the inclusion of children's sex and age, with previous literature establishing both sex and age as relevant factors impacting speech perception in noise [24, 25, 26]. Socioeconomic status was evaluated through the inclusion of maternal educational level (primary, secondary, or higher education) and household income (population tertiles) [23, 27, 28]. Furthermore, the impact of bilingualism on performance in speech‐in‐noise tests was considered, as previous research has suggested differential outcomes for individuals who are bi‐ or multilingual [29, 30, 31, 32]. Lastly, reported attention deficit problems were accounted for, measured by the Child Behavior Checklist (CBCL), given their potential influence on direct test performance [33].

### 2.7 Statistical Analysis

Individual and mean performance in the DIN test were computed. Linear regression models were utilized to examine the correlation between SRT concerning speech perception in noise and OM history, adjusted for confounders at the age of 9 and 13 years. Normality and homoscedasticity of the data, along with multicollinearity of the incorporated variables, were assessed for all the models. Missing data pertaining to the covariates were handled through the utilization of multiple imputation. Linear mixed‐effects models were employed to assess changes in DIN outcomes over time while accounting for the correlation between repeated measurements of each patient. Within the fixed‐effects part, we allowed for a nonlinear time effect using natural cubic splines with two internal knots placed at the respective percentiles of the follow‐up times. In the random‐effects part, we included random intercepts and random nonlinear splines utilizing the same splines as in the fixed‐effects part. The most suitable structure, tailored to the data, was determined through likelihood ratio tests. Additionally, residual plots were utilized to confirm the assumptions of the models. These analyses were conducted utilizing R software version 3.4.2. The study was approved by the Medical Ethical Committee of the Erasmus MC, University Medical Centre in Rotterdam. Written informed consent was obtained from all participants.

## 3 Results

**FIGURE 1:**
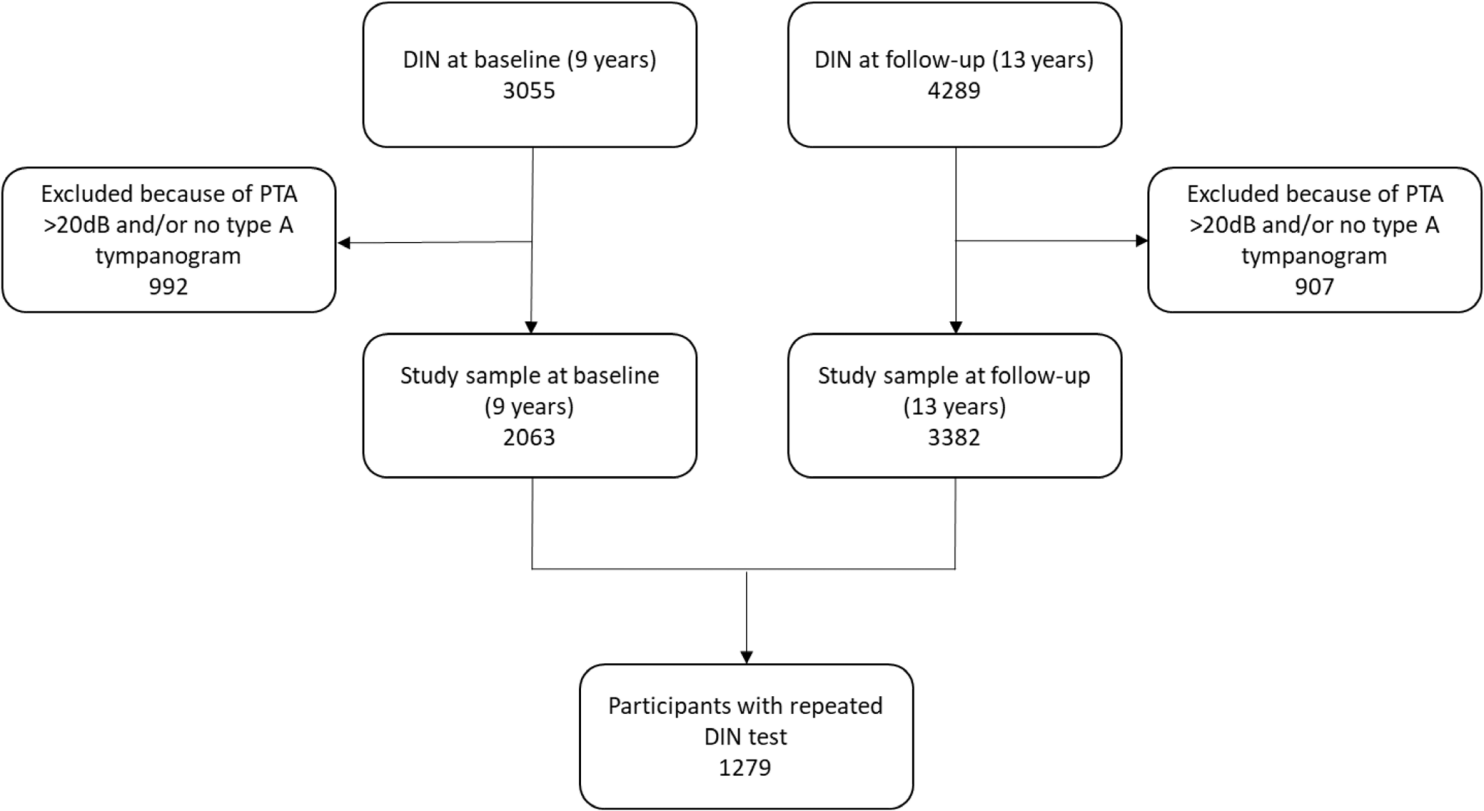
Flowchart of the study sample.

**FIGURE 2:**
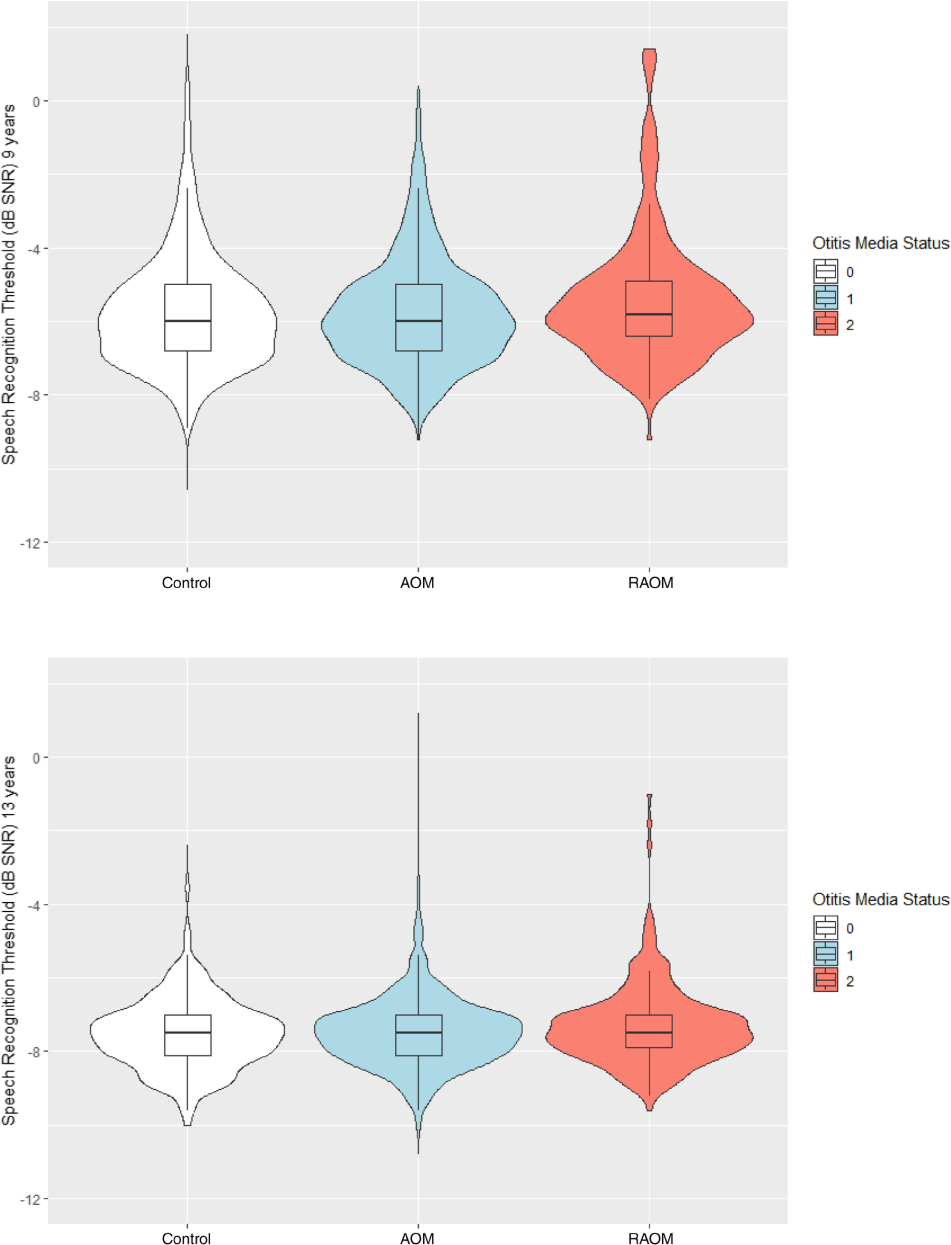
Violin plot analysis for SRT at baseline (9 years) and follow‐up (13 years) in OM groups. Violin plots represent kernel probability density, boxes represent interquartile ranges (with median and mean as solid and broken lines, respectively), and whiskers represent 1.5 times the interquartile range.

A total of 2063 children were included in the cross‐sectional analysis at baseline (9 years) with a mean age of 9.8 years old (ranged 8 years and 7 months to 12 years old), and an even distribution between girls (49.1%) and boys (50.9%). The mean SRT as measured for the better ear in the participants was −5.6 (SD = 2.0). In the cross‐sectional analysis at follow‐up (13 years), a total of 3382 children were included with a mean age of 13.6 years old (ranged 12 years and 6 months to 17 years old), and an even distribution between girls (49.7%) and boys (50.3%). The mean SRT as measured for the better ear in the participants at 13 years was −7.4 (SD = 1.4), which indicates better hearing performance compared to the baseline score of −5.6. A total of 1279 participants had data available for both measurement time points (9 and 13 years). Table 1 shows the characteristics of the study sample. Figure 1 shows a flowchart of the final study samples. Figure 2 shows violin plot analyses for SRT at baseline (9 years) and follow‐up (13 years) in OM groups.

**TABLE 1:** Characteristics of study sample at baseline (9 years) and follow‐up (13 years).

Characteristics	Study sample at baseline		Study sample at follow‐up	
No. of participants	2063		3382	
Sociodemographic characteristics	No. (%)	Mean (SD)	No. (%)	Mean (SD)
Age, years		9.8 (0.4)		13.6 (0.4)
Sex				
Male	1050 (50.9)		1701 (50.3)	
Female	1013 (49.1)		1681 (49.7)	
Maternal educational level				
No or primary	134 (6.5)		137 (4.0)	
Secondary	793 (38.4)		893 (26.4)	
Higher	953 (46.2)		1190 (35.2)	
Missing	183 (8.9)		1162 (34.4)	
Household income, tertile				
Lowest	238 (11.5)		253 (7.5)	
Middle	205 (9.9)		219 (6.5)	
Highest	1138 (55.2)		1440 (42.6)	
Missing	482 (23.4)		1470 (43.5)	
Multilingualism				
No	1000 (48.5)		2043 (60.4)	
Yes	300 (14.5)		570 (16.9)	
Missing	763 (37.0)		769 (22.7)	
Reported attention problem score		3.2 (3.2)		3.4 (3.1)
History of otitis media				
No	798 (38.7)		1206 (35.7)	
Yes	943 (45.7)		1299 (38.4)	
Recurrent	213 (10.3)		301 (8.9)	
Missing	109 (5.3)		576 (17.0)	
Hearing acuity				
SRT (dB SNR)		−5.6 (2.0)		−7.4 (1.4)

A multivariable linear regression analysis was conducted at both time points to investigate the cross‐sectional relationship between the DIN test and various literature‐based variables, including a history of OM (Table 2). The presence of a history of RAOM demonstrated a significant association with unfavorable outcomes on the DIN test at baseline (9 years) (*β* = 0.71; 0.20, 1.23; *p* = 0.007). Subsequently, a significant association persisted during the follow‐up (13 years) assessment (*β* = 0.21; 0.01, 0.41; *p* = 0.042). Furthermore, a statistically significant association between female sex and better outcomes on the DIN test (*β* = −0.13; −0.26, −0.01; *p* = 0.041) emerged at follow‐up (13 years). No significant associations were found with other covariates.

**TABLE 2:** Associations with SRT for data at baseline (9 years) and follow‐up (13 years).

Determinant	Multivariable analysis 9 years		Multivariable analysis 13 years	
	Beta coefficient (95% CI)	*p*	Beta coefficient (95% CI)	*p*
Sex				
Male	Reference		Reference	
Female	−0.24 (−0.55, 0.07)	0.135	−0.13 (−0.26, −0.01)	**0.041**
Maternal educational level				
No or primary	Reference		Reference	
Secondary	−0.48 (−0.47, 1.43)	0.328	0.07 (−0.35, 0.48)	0.758
Highest	0.46 (−0.52, 1.43)	0.359	0.03 (−0.39, 0.45)	0.884
Household income, tertile				
Lowest	Reference		Reference	
Middle	0.13 (−0.58, 0.84)	0.715	0.05 (−0.23, 0.33)	0.746
Highest	−0.17 (−0.77, 0.42)	0.572	0.08 (−0.14, 0.30)	0.483
Multilingualism				
No	Reference		Reference	
Yes	0.17 (−0.22, 0.57)	0.390	0.04 (−0.13, 0.20)	0.673
Reported attention problems	0.03 (−0.03, 0.07)	0.475	0.02 (−0.00, 0.04)	0.121
History of otitis media				
No	Reference			
Yes	0.01 (−0.32, 0.34)	0.939	0.03 (−0.11, 0.16)	0.676
Recurrent	0.71 (0.20, 1.23)	**0.007**	0.21 (0.01, 0.41)	**0.042**

*Note:* Bold values indicate statistical significance (*p* value < 0.05).

Post hoc analyses were conducted to evaluate the effect sizes for SRT across the three groups (control, OM, and RAOM) at both 9 and 13 years. At 9 years, Cohen's *d* values indicated a small to moderate effect between the control and RAOM group (−0.30) and a similar effect between the OM and RAOM group (−0.26). At 13 years, the effect sizes remained consistent, with Cohen's *d* for the control and RAOM group at −0.30 and for the OM and RAOM group at −0.28.

An additional linear mixed model was conducted to explore the relationship between repeated DIN tests at ages 9 and 13 within the same cohort for the longitudinal analysis (Table 3). The findings revealed that, after adjusting for other variables, the group with a history of RAOM demonstrated a statistically significant positive association with the dependent DIN test. The estimated coefficient was 0.552, with a standard error of 0.15 and a *t*‐value of 3.70 (*p* = < 0.001). These findings indicate that a history of RAOM significantly influences DIN test outcomes, leading to higher DIN scores, while keeping other covariates constant. Furthermore, there was a significant effect of sex on the dependent variable DIN. The estimated coefficient for girls was −0.21, with a standard error of 0.09 and a *t*‐value of −2.44 (*p* = 0.015). This indicates that sex also has a significant effect on DIN test outcome, with the DIN score being better for the girls. The remaining covariates did not manifest any statistically significant associations. Table 4 displays the generalized variance inflation factor (GVIF) values pertaining to multicollinearity.

**TABLE 3:** Linear mixed model.

	Estimate	95% CI	*t*	*p*
Intercept	−7.07	−9.11, −5.03	−6.80	**< 0.001**
Sex (girl)	−0.21	−0.39, −0.04	−2.44	**0.015**
Maternal educational level (secondary)	0.41	−1.67, 2.49	0.39	0.698
Maternal educational level (higher)	0.35	−1.74, 2.43	0.33	0.745
Household income (middle)	0.13	−0.55, 0.82	0.38	0.706
Household income (highest)	0.01	−0.64, 0.67	0.04	0.967
Multilingualism (yes)	0.15	−0.07, 0.38	1.32	0.188
Attention problems	−0.00	−0.03, 0.03	−0.05	0.960
Otitis media	0.08	−0.10, 0.26	0.85	0.397
Recurrent otitis media	0.55	0.26, 0.84	3.70	**< 0.001**

*Note:* Bold values indicate statistical significance (*p* value < 0.05).

**TABLE 4:** Generalized variance inflation factor (GVIF).

Variable	GVIF
Sex	1.03
Maternal educational level	1.31
Household income	1.38
Multilingualism	1.13
Attention problems	1.04
Otitis media status	1.01

## 4 Discussion

The results of the current study provide new evidence indicating that RAOM is associated with subsequent deficits in auditory functioning, as measured by speech perception in noisy environments, in children aged 9–13 years old. We found significantly poorer outcomes in speech recognition in noise among children with a documented history of RAOM. Notably, all participants had normal audiometric thresholds (≤ 20 dB HL) and type A tympanograms at the time of testing.

A few studies have demonstrated diminished signal detection and discrimination in noisy conditions that persist after the resolution of OM [12, 13, 14, 15, 16]. Mishra et al. provide a unique perspective by investigating auditory deprivation during development and its influence on efferent neural feedback and perception [17]. Understanding alterations in neural feedback mechanisms enriches our comprehension of how OM may shape the auditory system. Although the magnitude of the difference in efferent inhibition between the OM and control group was small, they suggest that a statistically small effect size can have important physiological consequences. For instance, a small efferent effect is associated with a relatively larger release from adaptation in the auditory‐nerve response to noisy speech, resulting in enhanced speech intelligibility [34].

Gravel et al. found that the extended high‐frequency hearing (12.5, 14, and 16 kHz) and brain stem auditory pathway measures of 8‐year‐old children were significantly influenced by a history of OM [35]. It is possible that inflammatory toxins could adversely impact the inner ear, particularly affecting higher frequencies due to their positioning at the base of the cochlea and proximity to the inflammatory process of the middle ear [6, 7]. In contrast with our findings, another study by Gravel et al. found no long‐term peripheral hearing loss after OME had resolved, but their follow‐up was limited to age 3. Our findings suggest that the impact may extend beyond peripheral measures, as we followed children at ages 9 and 13 years [36]. Congruent with earlier findings in the study of Gravel and Wallace of 4‐year‐old children, our study supports the notion that recovery of auditory skills remains incomplete even up to school age [37]. The results establish a correlation between middle ear history and diminished performance in speech perception in noise, persisting until the age of 13 years. Impaired speech perception in noise may adversely impact daily situations and hinder communication, particularly in educational environments with background noise.

Further supporting these findings, recent research by Nittrouer and Lowenstein (2024) showed that children with a history of early OM exhibited poorer performance in both auditory and language tasks, with the effects being stronger for phonological sensitivity than for lexical knowledge [38]. These results emphasize the potential for long‐term auditory deficits following early OM, especially affecting the ability to process complex auditory stimuli, which may contribute to challenges in speech recognition. This aligns with our findings, where children with a history of RAOM showed persistent difficulties in speech perception in noise, despite normal audiometric thresholds at the time of testing.

It is important to note that interventions such as tympanostomy tube insertion or antibiotic treatments may play a significant role in mitigating the long‐term effects of RAOM on speech perception. According to a meta‐analysis by Steele et al. (2017), tympanostomy tubes improve hearing in the short‐term and may reduce the frequency of RAOM episodes [39]. Although the long‐term benefits on auditory function, such as speech perception in noise, remain unclear, this intervention could play an important role in reducing the cumulative effects of recurrent infections on auditory development.

The findings from this study indicate that RAOM is not only a temporary condition, but may also contribute to ongoing challenges in speech recognition, particularly in noisy environments. Given the significant effect of RAOM on speech perception in noise, it may be beneficial for healthcare practitioners and educators to remain attentive to the auditory development of children with a history of RAOM, incorporating comprehensive assessments that extend beyond the resolution of acute symptoms. While the observed effect sizes are small to moderate, they highlight the potential for persistent challenges in speech recognition. However, their clinical relevance may be less apparent than the statistical significance might initially suggest.

## 5 Strengths and Limitations

A strength of this study lies in its prospective design and substantial sample size. The utilization of a linear mixed model within a large cohort provides a robust analytical framework, accommodating correlations between repeated measures. However, a limitation of the study arises from the reliance on questionnaires rather than otoscopic examination for determining the OM status. Additionally, information regarding the duration of OM episodes, use of antibiotics, and any tympanostomy‐related treatments was not available. Unfortunately, no distinction could be made between unilateral or bilateral ear infections from the questionnaires. The cohort's selection bias toward a predominantly highly educated population might impact the generalizability of our findings over time. Ultimately, the effect size appears to be small to moderate, suggesting that while our findings are significant, they may have limited clinical impact.

## 6 Conclusions

Our findings from this longitudinal study suggest a potential association between a prior history of RAOM and elevated DIN test scores up to the age of 13 years. While this association appears to persist over time, the effect sizes observed were small to moderate. Further investigation is needed to clarify whether this relationship continues into later stages of life and to better understand its clinical significance.

## Disclosure

The study sponsors had no role in the study design, data analysis, interpretation of data, or writing of this report.

## Conflicts of Interest

The authors declare no conflicts of interest.
